# Population genetic analysis of clinical *Mycobacterium abscessus* complex strains in China

**DOI:** 10.3389/fcimb.2024.1496896

**Published:** 2025-01-20

**Authors:** Xiangchen Li, Yelei Zhu, Yewei Lu, Kunyang Wu, Yang Che, Xiaomeng Wang, Weixin Wang, Junli Gao, Junshun Gao, Zhengwei Liu, Zhuxian Zhou

**Affiliations:** ^1^ College of Chemical and Biological Engineering, Zhejiang University, Hangzhou, Zhejiang, China; ^2^ Shaoxing Key Laboratory of Infectious Diseases, Affiliated Hospital of Shaoxing University, Shaoxing, Zhejiang, China; ^3^ The Institute of TB Control, Zhejiang Provincial Center for Disease Control and Prevention, Hangzhou, Zhejiang, China; ^4^ Institute of Tuberculosis Prevention and Control, Ningbo Municipal Center for Disease Control and Prevention, Ningbo, Zhejiang, China; ^5^ Key Laboratory of Vaccine, Prevention and Control of Infectious Disease of Zhejiang Province, Zhejiang Provincial Center for Disease Control and Prevention, Hangzhou, Zhejiang, China

**Keywords:** mycobacterium abscessus complex, population Genetics, molecular epidemiology, cgMLST, pan-genome

## Abstract

**Background:**

To explore the genetic characteristics of the *Mycobacterium abscessus* complex (MABC) population in China, given its rising clinical importance among nontuberculous mycobacteria.

**Methods:**

We conducted population genetic analyses on 360 MABC genomes from China, focusing on core genome multilocus sequence typing (cgMLST), pan-genome characterization, population genetics, and antimicrobial resistance gene profiling.

**Results:**

Our analysis identified 273 *M. abscessus* subsp. *abscessus* (Mab_A_) and 87 *M. abscessus* subsp. *massiliense* (Mab_M_) isolates, uncovering 68 sequence types (STs), with ST5 being the most common. cgMLST classified 33.3% of isolates into six dominant circulating clones (DCCs) and 49.4% into 59 genomic clusters at a threshold of 25 different alleles, including 18 international clusters linking Chinese isolates with seven other countries. The MABC pan-genome is open, with Mab_A_ exhibiting greater accessory gene diversity and higher gene turnover compared to Mab_M_. Mobile genetic elements (MGEs), such as prophages and genomic islands, were prevalent across all genomes. 139 to 151 virulence factors (VFs) were identified per genome, with distinct accessory VFs in Mab_A_ and Mab_M_ affecting immune modulation and metabolism. Resistance gene profiling revealed ubiquitous *mtrA*, *RbpA*, and *bla*_MAB_, with Mab_A_-specific *erm(41)* conferring resistance to macrolides and β-lactams. Common *rrs* and *rrl* gene mutations indicated widespread resistance to aminoglycosides and macrolides, while *gyrA* mutations suggested emerging fluoroquinolone resistance. An acquired *erm(46)* gene, likely obtained via phage-mediated horizontal gene transfer, was detected in one Mab_A_ strain.

**Conclusion:**

This study provides key genetic insights into the dynamics of MABC in China. The widespread distribution of DCCs, high genomic clustering rates, open pan-genome, and distinct resistance patterns between Mab_A_ and Mab_M_, along with MGEs, highlight the need for targeted surveillance and tailored therapies to address emerging challenges in MABC infections.

## 1 Introduction

The *Mycobacterium abscessus* complex (MABC) is a major group of nontuberculous mycobacteria (NTM) that has recently garnered increasing attention due to the rising incidence of infections worldwide (Johansen et al., 2020). MABC encompasses a high degree of genomic diversity, comprising three subspecies: *M. abscessus* subsp. *abscessus*, *M. abscessus* subsp. *bolletii*, and *M. abscessus* subsp. *massiliense*, commonly referred to as *M. abscessus* (Mab_A_), *M. bolletii* (Mab_B_), and *M. massiliense* (Mab_M_), respectively (Bryant et al., 2021). These mycobacteria are ubiquitous in various environments, including water, soil, and dust (Falkinham, 2009). Transmission typically occurs indirectly via fomite contamination or persistent infectious aerosols; however, person-to-person transmission has also been reported, particularly among cystic fibrosis patients, who are more susceptible due to their compromised lung function and frequent hospitalizations (Bryant et al., 2013).

Recently, MABC strains have been increasingly implicated in pulmonary infections, complex skin and soft tissue infections, and disseminated diseases with often poor prognoses (Johansen et al., 2020). Notably, MABC accounts for 22.5% of all NTM clinical isolates in China, and the prevalence of NTM infections among suspected tuberculosis cases has reached 6.3% (Yu et al., 2016; Zhou et al., 2020). A recent study in Shanghai found that MABC was responsible for 16.2% of nontuberculous mycobacterial pulmonary disease (NTM-PD) cases, underscoring its significant prevalence and geographic variability in the region (Zhang et al., 2024).

Although MABC was originally thought to only be independently acquired from the environment, these isolates have evolved from environmental organisms into true pulmonary pathogens (Lopeman et al., 2019). Currently, over 70% of infections in CF patients are caused by genetically clustered isolates, the majority of which belong to seven dominant circulating clones (DCCs) (Bryant et al., 2016; Ruis et al., 2021). These DCCs, first emerging around 1960, have been linked to higher virulence, increased resistance, and worse clinical outcomes compared to unclustered isolates (Bryant et al., 2021).

Whole-genome sequencing (WGS) has proven invaluable for molecular outbreak investigations, source tracking, and population structure analysis, as evidenced by studies in the UK, Germany, and Portugal (Lipworth et al., 2021; Wetzstein et al., 2022; Carneiro et al., 2023). To support standardized molecular surveillance, Diricks et al. developed a core genome multilocus sequence typing (cgMLST) scheme for MABC, linking DCCs to a reference genome with fewer than 250 alleles, and showing that 99% of pairwise comparisons between epidemiologically related isolates had fewer than 25 alleles, with 90% under 10 alleles (Diricks et al., 2022).

In China, WGS-based research on MABC has primarily focused on antibiotic resistance mechanisms (Ye et al., 2019; Jin et al., 2022; Yang et al., 2024). While these studies have provided valuable insights into the genetic basis of drug resistance, there is a notable lack of research into the population genetics, pan-genome, and molecular epidemiology dynamics of MABC. Understanding these aspects is crucial for implementing effective public health interventions and controlling the spread of this pathogen.

This study aims to fill this gap by analyzing the population genomics of MABC in China using publicly available genome sequences. We implemented cgMLST to identify DCCs and detect potential transmission events. Additionally, we explored genetic diversity through population genetics, pan-genome analyses, and profiling of virulence factors (VFs), antimicrobial resistance genes (ARGs), and mobile genetic elements (MGEs). This work would enhance the understanding of MABC population genetic diversity in China, thereby informing public health strategies and clinical management.

## 2 Materials and methods

### 2.1 Genome collection, quality control and characterization

We retrieved all publicly available MABC genome assemblies from the Bacterial and Viral Bioinformatics Resource Center (BV-BRC) as of May 28, 2024, using the search criteria: “GENOMES = Mycobacterium abscessus,” “genome status = WGS,” and “genome quality = good.” The BV-BRC database was chosen for its extensive and high-quality collection of publicly accessible genomic sequences and other omics-related data for bacterial research, rendering it an optimal choice for population genomics studies (Olson et al., 2023). BV-BRC defines “good” genome quality based on criteria such as high-quality sequencing data, minimal contamination, and a complete genome assembly with high coverage, ensuring reliable downstream analysis. All strains were of human origin. Corresponding metadata were obtained from the BV-BRC and cross-verified with the NCBI GenBank database (**Supplementary Table S1**). The strains were classified into Chinese and Global strains based on the “Isolation Country” information in the metadata. Additionally, we included raw WGS data from two Chinese MABC strains (NCBI SRA: SRR28113991 and SRR26200315) sourced from recent studies (Liao et al., 2024; Peng et al., 2024). The raw reads underwent quality control using fastp and genome assembly with Unicycler, both with default settings (Wick et al., 2017; Chen et al., 2018).

We assessed the quality of the genome assemblies using QUAST v5.0.2, CheckM v1.1.3, and fastANI v1.34 (Gurevich et al., 2013; Parks et al., 2015; Jain et al., 2018). All genomes met the quality criteria with contamination levels below 5%, completeness over 90%, and an average nucleotide identity (ANI) greater than 98% compared to the reference genome GZ002 (RefSeq: NZ_CP034181.1).

### 2.2 Genome annotation

We annotated all MABC genomes using Prokka v1.14.5 with default parameters. ARGs were identified via RGI v6.0.3, referencing the CARD database v3.2.9 (Alcock et al., 2023). VF genes were screened using the VFDB database (Liu et al., 2022). Prophages were detected and extracted with Phigaro (Starikova et al., 2020). Plasmid content was reconstructed using MOB-suite v3.0.3, specifically MOB-recon, which included relaxase and replicon typing, and generated MOB-cluster codes and host range data (Robertson and Nash, 2018). The identification and characterization of insertion sequence (IS) elements, down to the family level, were performed using digIS (Puterová and Martínek, 2021). Integrative and conjugative elements (ICEs) were identified with ICEberg v2.0 (Liu et al., 2019). Genome visualization was performed using the Proksee website (Grant et al., 2023).

### 2.3 Molecular genotyping and genomic clustering

Assemblies were compared to a reference sequence for each of the three subspecies, and whole-genome ANI scores were calculated using fastANI. Our reference genomes were ATCC19977 (NCBI RefSeq: NC_010397) for Mab_A_, GO06 (NC_018150) for Mab_M_, and FLAC003 (NZ_CP014950) for Mab_B_. Genomes were assigned to a subspecies based on an ANI of at least 98% with one reference strain (Commins et al., 2023). The specific sequence type (ST) of each genome was identified with pyMLST v2.1.6 using PubMLST typing schemes (Jolley et al., 2018; Biguenet et al., 2023).

The core genome multilocus sequence typing (cgMLST) allelic profiles were determined using chewBBACA v3.1.2 based on a 2,904-loci schema from the RIDOM Nomenclature Server (https://www.cgmlst.org/) developed by Diricks and colleagues (Silva et al., 2018; Diricks et al., 2022). Pairwise cgMLST distances were generated using cgmlst-dists v0.4.0 based on the core genome genes present in more than 95% of the collected genomes. The 95% threshold was chosen to ensure that only core genes, which are highly conserved and present in the majority of isolates, were included in the analysis, thereby minimizing the influence of strain-specific variations and ensuring robust and reliable distance calculations. A minimum spanning tree (MST) was constructed using GrapeTree v1.5.0 with the MSTv2 algorithm (Zhou et al., 2018).

### 2.4 Phylogenetic reconstruction

Core genome alignment and SNP calling (cgSNP) were performed with Parsnp v1.2 from the HarvestTools kit, using GZ002 as the reference (Treangen et al., 2014). Phylogenetic trees were then constructed using RAxML v8.2.9 with the core genome SNP alignment after predicted recombination sites were removed by Gubbins v2.1.0 (Stamatakis, 2014; Croucher et al., 2015). We used a general-time reversible nucleotide substitution model with a GAMMA correction for site variation and applied 1,000 bootstrap replicates with Lewis ascertainment correction for tree construction. Phylogenetic trees were visualized using the Interactive Tree of Life (iTOL) website (Letunic and Bork, 2021).

### 2.5 Pangenome investigation

The pangenome was reconstructed using Panaroo v1.5.0 in “strict” mode to filter out potential contamination and erroneous annotations (Seemann, 2014; Tonkin-Hill et al., 2020). The resulting gene presence-absence matrix, along with associated metadata, was analyzed in R using the Pagoo framework for principal component analysis (PCA) and to define pangenome structure (Ferrés and Iraola, 2021). The R package micropan was employed to estimate pangenome size and openness according to Heap’s law model and to compute genomic fluidity (Snipen and Liland, 2015). Gene gain and loss rates between different subspecies were calculated using Panstripe v0.3 (Tonkin-Hill et al., 2023).

### 2.6 Population genetic analysis

Core genes from each subspecies, as inferred by Panaroo, were aligned using MAFFT v7.525 (Katoh and Standley, 2013). Nucleotide diversity ( π ) and Tajima’s *D* values for each core gene were calculated with the R package pegas (Paradis, 2010). Genome alignments between subspecies, constructed from Parsnp, were input into ClonalFrameML v1.13 to infer the recombination rate ( R/θ ), the ratio of recombination to mutation rates ( R/m ), and the locations of recombination sites within and between subspecies (Didelot and Wilson, 2015).

## 3 Results

### 3.1 Overview of Chinese MABC isolates

In this study, we obtained 360 MABC genome assemblies from China, following a database screening on May 28, 2024. These isolates, collected between 2018 and 2021, came from twelve studies across eight provinces and municipalities (**Supplementary Table S1**). The isolates were all clinically relevant, with the majority (93.9%, 338/360) derived from *in vitro* culture of pulmonary samples. Shanghai had the highest number of isolates, representing 89.2% ( n=321 ) of the total, followed by Zhejiang Province with 6.1% ( n=22 ), while other regions contributed only a small number of isolates.

### 3.2 Phylogenetic structure of Chinese MABC isolates

Molecular genotyping using ANI identified 273 isolates (75.8%) as Mab_A_ and 87 (24.2%) as Mab_M_ (**Supplementary Table S2**). MLST analysis based on the scheme from PubMLST database revealed 68 distinct STs among 350 isolates, with 11 STs having more than 10 members. ST5 was the most predominant ( n=63 ), followed by ST3 ( n=46 ). Additionally, 34 STs were represented by only a single isolate each. We further applied the cgMLST scheme developed by Diricks et al., using a threshold of 250 allelic differences (ADs) for DCC classification (Diricks et al., 2022) and identified six DCCs, comprising 120 (33.3%) Chinese isolates (**Supplementary Table S3A**).

**Figure 1:**
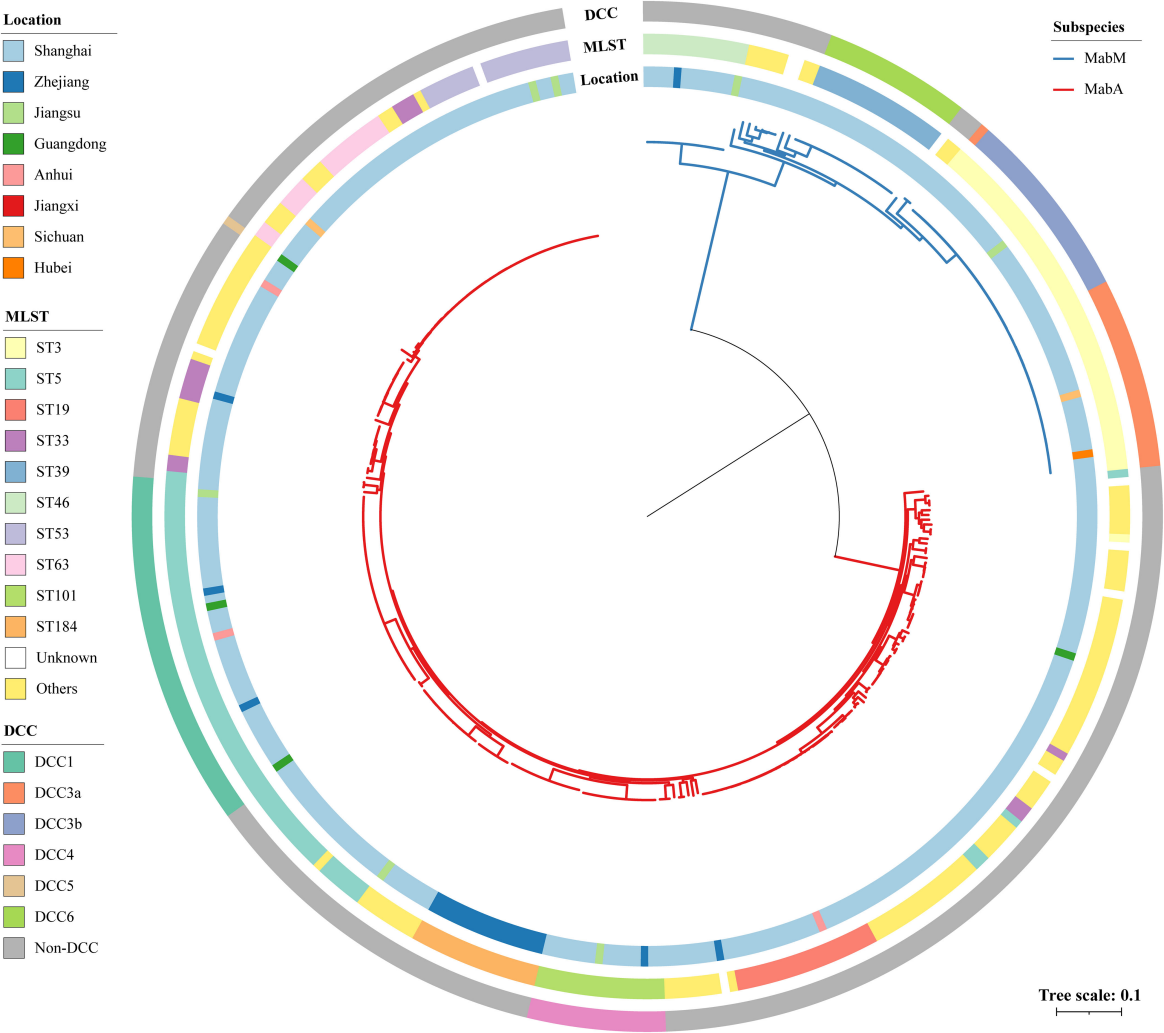
Phylogeny and genotype of 360 Chinese MABC isolates. The different colors on the branches indicate different subspecies. The inner ring represents the collection location, the middle ring shows the STs based on the 7-loci MLST scheme hosted on PubMLST database, and the outer ring indicates the DCC clades. Scale bar shows the number of substitutions per site.

To refine the evolutionary relationships among Chinese MABC, we reconstructed a phylogenetic tree for all 273 isolates, mapping it with ST, collection location and DCC (**Figure 1**). Phylogenetic analysis revealed a clear divergence between Mab_A_ and Mab_M_. The terminal branch lengths, reflecting the degree of genetic divergence and accumulated evolutionary changes, were significantly shorter for Mab_A_ isolates compared to Mab_M_ isolates (**Supplementary Figure S1**).

Mab_A_ showed greater genetic diversity than Mab_M_, with 58 STs dominated by ST5 (23.1%, 63/273), while Mab_M_ has 11 STs, primarily ST3 (51.8%). The proportion of DCC strains was higher in Mab_M_ (71.3%, 62/87) than in Mab_A_ (21.2%, 58/273). Mab_M_ DCC strains were further divided into DCC3a (36.1%, 22/62), DCC3b (36.1%, 22/62), and DCC6 (27.9%, 17/62). In Mab_A_, DCC strains were classified into DCC1 (70.7%, 41/58), DCC42 (27.6%, 16/58), and DCC5 (1.7%, 1/58). Additionally, strains belonging to DCCs exhibit tighter clustering than those belonging to STs on the tree.

### 3.3 Genomic clustering for Chinese MABC isolates

**Figure 2:**
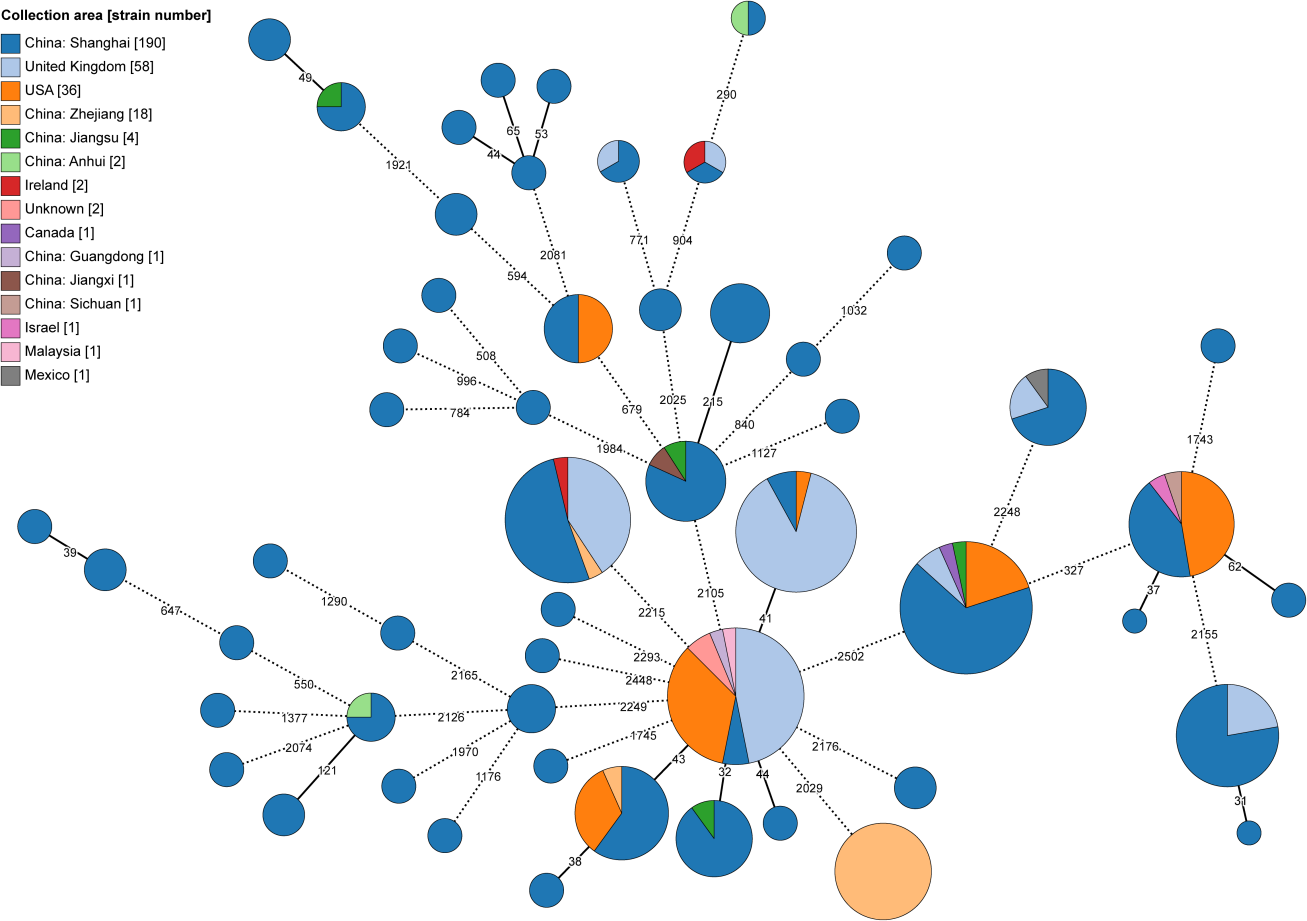
Minimum-spanning tree of recent transmission clustered MABC isolates based on the cgMLST. The size of each node corresponds to the number of isolates within a cluster, with each node’s color representing the isolation area shown as a pie chart. Nodes with fewer than 25 ADs were collapsed together, and branches longer than 250 ADs were shortened and indicated with a hashed line.

Using a 25 AD threshold for potential recent transmission, as suggested by Diricks et al (Diricks et al., 2022), we grouped 178 (49.4%) of the Chinese isolates into 59 genomic clusters, ranging from 2 to 16 isolates per cluster (**Figure 2**; **Supplementary Table S3B**). The clustering rate for Mab_A_ was 50.2% (137/273), slightly higher than for Mab_M_ at 47.1% (41/87). The largest genomic cluster comprised 16 clinical isolates collected in January 2016 from a hospital in Zhejiang Province, including 14 from sputum samples and 4 from ventilator condensate. Previous studies indicated these isolates were linked to a pulmonary MABC outbreak among elderly ICU patients (Hua et al., 2023). Our cgMLST analysis supported this, showing pairwise distances ranging from 2 to 20 ADs, consistent with recent transmission.

The remaining 162 clustered isolates were reported from four NCBI BioProjects linked to a pulmonary hospital in Shanghai. Among these, 69 isolates were part of DCC clusters, with 23 in DCC1, 17 in DCC3b, 13 in DCC4, 10 in DCC6, and 6 in DCC3a. The samples were collected between 2014 and 2017, from locations including Shanghai, Jiangsu, Zhejiang, Anhui, and Jiangxi Provinces—all in Eastern China.

Additionally, we identified 18 clusters at a 25 AD threshold, comprising 65 Chinese isolates and 100 global isolates from seven other countries retrieved from the BV-BRC database (**Supplementary Tables S3C, D**). These clusters included 58 isolates from the UK, 36 from the USA, and others from Ireland ( n=2 ), Canada ( n=1 ), Israel ( n=1 ), Malaysia ( n=1 ), and Mexico ( n=1 ).

### 3.4 Evolutionary characteristics and nucleotide diversity of Mab_A_ and Mab_M_ isolates

**Figure 3:**
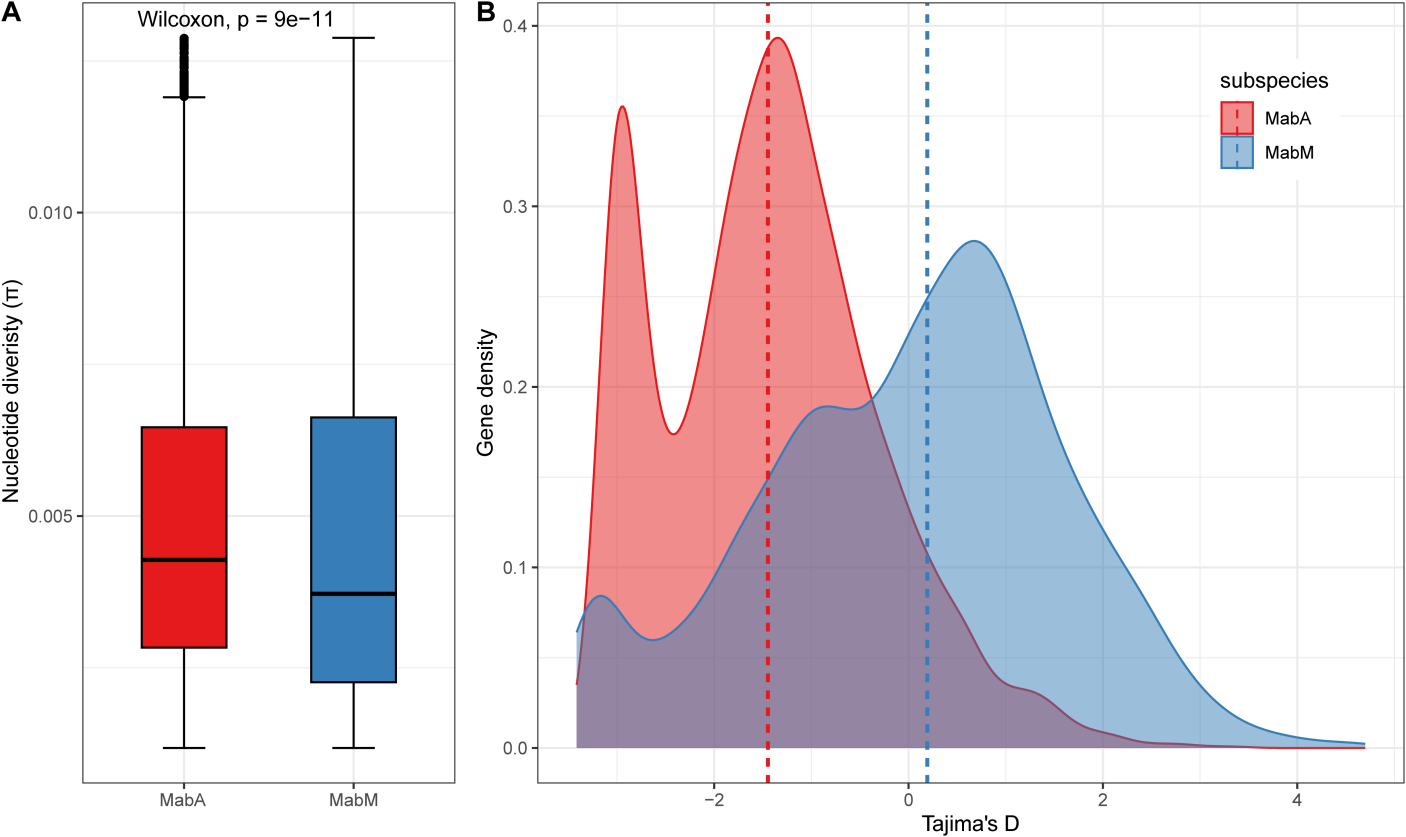
Population genetics of Mab_A_ and Mab_M_. **(A)** Nucleotide diversity (π) values distribution. **(B)** Tajima’s D values distribution. The dashed line represents the median.

To discover the evolutionary characteristics of Mab_A_ and Mab_M_, we measure nucleotide diversity per site ( π ) and Tajima’s *D* values based on the core genomes (**Figure 3**). Mab_A_ isolates (median $\pi =7.13e^{-3}$ ) was more diverse than Mab_M_ isolates (median $\pi =6.84e^{-3}$ ) in terms of SNP diversity of the core genome (Wilcoxon rank sum test, P-value< $2.2e^{-16}$ ). Furthermore, the Tajima’s *D* values for Mab_A_ isolates displayed a skewed distribution with a high proportion of negative values (median Tajima’s *D* = -1.42), indicating possible purifying selection or population expansion. In contrast, the distribution for Mab_M_ was more balanced around zero (median Tajima’s *D* = 0.21), suggesting a population at equilibrium. It is worth noting that sequencing depth, which ranged from 67× to 247× in this study, may influence the detection of rare variants, potentially affecting π and Tajima’s *D* values. However, as sequencing depths were consistently above 60× , their impact on overall results is expected to be minimal.

We also conducted ClonalFrameML analyses to estimate homologous recombination rates among the Chinese MABC isolates. Across both Mab_A_ and Mab_M_ subspecies, the rate of homologous recombination relative to mutation ( R/θ ) was less than one-half, with the recombination-to-mutation ratio ( R/m ) estimated at 2.02. This indicates that while recombination events are less frequent than mutations, their impact on nucleotide variation is twice as significant.

### 3.5 Pan-genome analysis reveals genomic diversity and flexibility among Chinese MABC isolates

**Figure 4:**
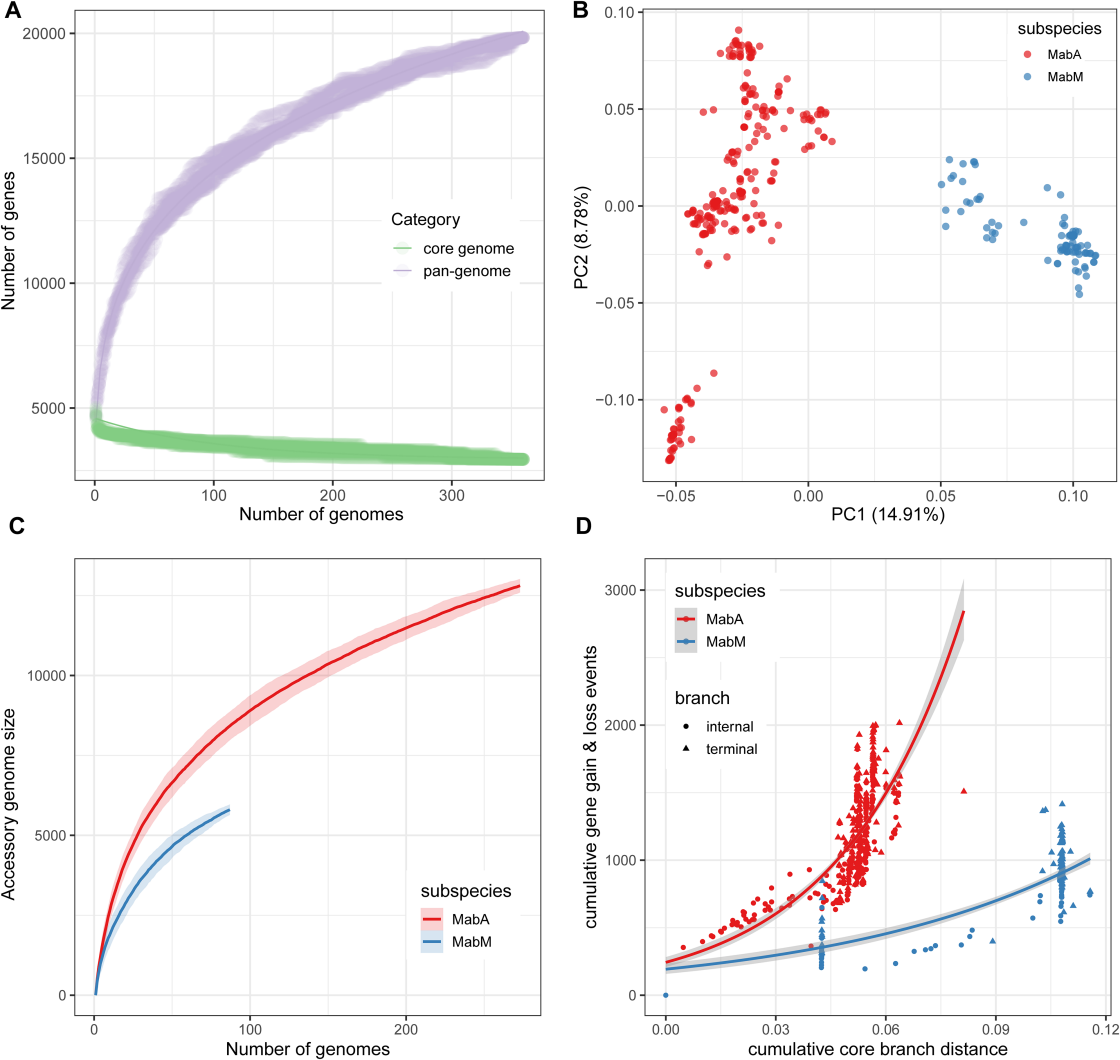
Characteristics of the Chinese MABC pan-genome. **(A)** Growth curves of the core and pan-genome of 360 MABC genomes. **(B)** Two-dimensional PCA visualization of the MABC pan-genome. **(C)** Growth curves of the accessory genomes for both Mab_A_ and Mab_M_. **(D)** The cumulative number of gene gain and loss events plotted against the cumulative branch length from the root node of each tree for Mab_A_ and Mab_M_. Internal branches are represented by circles, and terminal branches by triangles.

We conducted a pan-genome analysis to explore the genomic diversity among Chinese MABC isolates, categorizing genes into core and accessory groups. The analysis revealed a total pan-genome size of 19,830 genes, with 3,994 (20.1%) identified as core genes (>95% prevalence) and 15,836 (79.9%) as accessory genes (**Figure 4A**). Heaps’ law modeling ( $n\;=\;\kappa N^{\lambda }$ ) of the gene presence-absence matrix produced a γ value of 0.53, indicating an open pan-genome (Vernikos et al., 2015). This suggests that the pan-genome continues to expand as new genes are introduced, without reaching saturation.

Principal component analysis (PCA) based on gene presence-absence data showed a clear separation between Mab_A_ and Mab_M_ genomes (**Figure 4B**), consistent with their subspecies classification derived from cgSNP-based phylogenetic analysis. The diversity in accessory genes was higher in Mab_A_ compared to Mab_M_ for a similar number of strains, highlighting greater genetic flexibility of Mab_A_ (**Figure 4C**). Despite this, the core genomes of Mab_A_ and Mab_M_ were similar, consisting of 4,141 and 4,067 genes, respectively. Further analysis using Panstripe, based on the cgSNP-based phylogeny and gene presence-absence matrix, revealed significantly higher rates of gene gain and loss in Mab_A_ compared to Mab_M_ (P-value = $1.29e^{-5}$ ). Notably, these gene gain and loss events were predominantly clustered in the more ancestral branches of the phylogenies for both subspecies (**Figure 4D**).

### 3.6 Distribution of MGEs in MABC genomes

We identified a total of 45 plasmids across 39 genomes. Among these, two genomes harbored three plasmids each, another two genomes contained two plasmids each, and the remaining 35 genomes each contained one plasmid (**Supplementary Table S4**). These plasmids were categorized into nine MOB-clusters (AA558, AB922, AB951, AE904, AG074, AG676, AG677, AG701, AG806), with AA701 being the most common (64.4%, n=29). Among them, three clusters (AA558, AE904, AG701) were predicted to be mobilizable due to the presence of either a relaxase or an *oriT*, but lacked the mate-pair formation marker. Furthermore, 125 ICEs were detected in 111 genomes, with each genome containing between 1 and 3 ICEs. Among them, 84% (n=105) belonged to Mab2101 of AICE. Notably, no ARGs or VFs were found on these plasmids or ICEs.

We uncovered 783 prophage sequences in 288 genomes, with each genome containing between 1 and 7 prophages, ranging in length from 0.66 kb to 210.9 kb. Taxonomic classification revealed that the majority of these prophages belonged to the *Siphoviridae* family ( n=699 ), followed by *Podoviridae* ( n=13 ) and *Myoviridae* ( n=3 ). Moreover, 700 ISs were predicted in 254 MABC genomes, primarily from the families IS*481* ( n=110 ), IS*256* ( n=85 ), IS*607* (*n* = 65), and IS*21* ( n=59 ). We also identified 1,303 genomic islands (an average of 3.7 ± 1.8 per genome) in 352 genomes, with total lengths per genome ranging from 5.5 kb to 289.5 kb.

### 3.7 Virulence and antimicrobial resistance genetic profiles

Across the 360 MABC genomes, we identified between 139 to 151 VFs, including 134 core VFs (present in more than 95% of all strains) and 17 accessory VFs (**Supplementary Table S5**). The accessory VF profiles of Mab_A_ and Mab_M_ were distinct, particularly in effector delivery systems, immune modulation, regulation, and nutritional/metabolic functions. Notably, immune modulation and nutritional/metabolic VFs displayed specific factors unique to each subspecies: *MAB4114* and *MAB_3999* in Mab_A_, and *MASS_RS20860* and *MASS_RS20355* in Mab_M_. The virulence gene *mce9D* was found in 87.4% (76/87) of Mab_M_ isolates but absent in Mab_A_ isolates. Similarly, *pcaA* was present in 67.8% (59/87) of Mab_M_ isolates and 2.2% (6/273) of Mab_A_ isolates.

In terms of antimicrobial resistance, the macrolide and penam resistance gene *mtrA*, along with the rifampin resistance-associated gene *RbpA*, were prevalent across all genomes analyzed (**Table 1**). The chromosomally encoded β-lactamase *bla*_MAB_, which contributes significantly to β-lactam resistance, was found in 349 genomes (**Supplementary Table S6**). The resistance gene *erm(41)*, associated with resistance to macrolide, streptogramin, and lincosamide, was prevalent only in Mab_A_ and absent in Mab_M_. Aminoglycoside resistance-related *rrs* gene mutations, predominantly n.1408A>G, were identified in 347 isolates, with additional mutations n.1355A>G and n.1375A>G found in 4 Mab_A_ isolates. The clarithromycin resistance-conferring *rrl* gene mutation n.2059A>G was present in 338 genomes, with three Mab_M_ strains also harboring the n.2271A>G mutation. Furthermore, fluoroquinolone resistance-related mutations in the *gyrA* gene (p.95D>G, p.90A>V, p.89D>N) were detected in four Mab_A_ isolates.

**Table 1:** Antibiotic resistance genes found among Chinese MABC isolates.

ARG	Mab_A_ ( n=273 )	Mab_M_ ( n=87 )	Mutation	Antibiotics	Mechanism of resistance
*mtrA*	273	87	–	penam, macrolide	antibiotic efflux
*RbpA*	273	87	–	rifamycin	antibiotic target protection
*bla*_MAB_	273	86		penem, penam, cephalosporin	antibiotic inactivation
*erm(41)*	273	0	–	streptogramin, macrolide, lincosamide	antibiotic target alteration
*erm(46)*	1	0	–		
*rrs*	271	86	n.1408A>G	aminoglycoside	antibiotic target alteration
	4	0	n.1355A>G		
	4	0	n.1375A>G		
*rrl*	266	82	n.2059A>G	macrolide	antibiotic target alteration
	0	3	n.2271A>G		
*gyrA*	2	0	p.95D>G	fluoroquinolone	antibiotic target alteration
	1	0	p.90A>V		
	1	0	p.89D>N		

**Figure 5:**
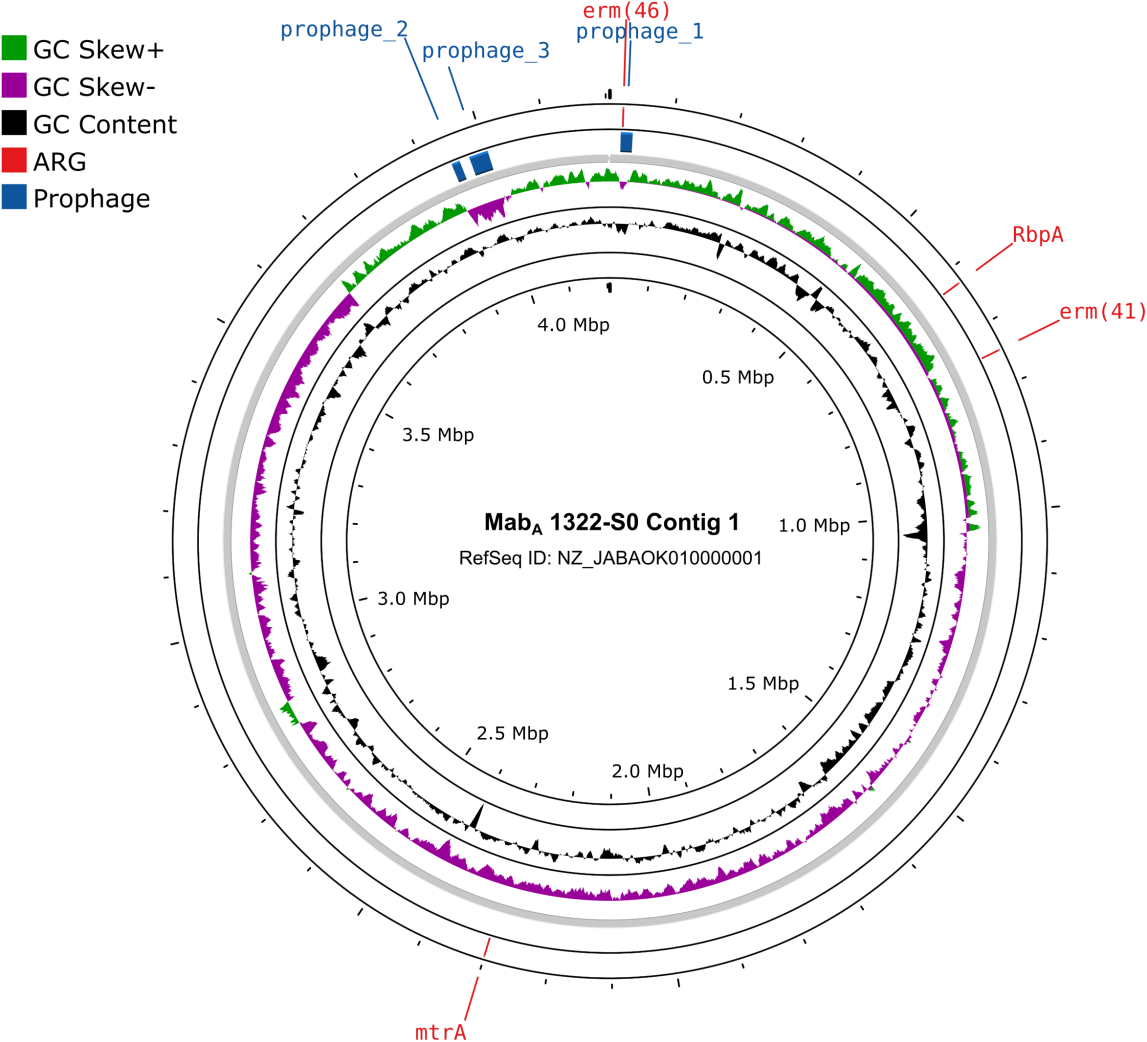
Circular representation of the MabA 1322-S0 contig 1. The circles in the plot, from the inside to the outside, represent genomic GC Content, genomic GC Skew, prophages and ARGs.

A notable finding was the identification of an acquired antimicrobial resistance gene *erm(46)*, conferring resistance to macrolide, streptogramin, and lincosamide, in one Mab_A_ isolate (1322-S0, GenBank: GCA_012845855). A BlastP comparison from NCBI NR database revealed a sequence identity and coverage of 100% with the *erm(46)* gene from *Rhodococcus equi* (WP_170092264). This gene was located on a 19.2-kb *Siphoviridae* prophage in Mab_A_ 1322-S0 (**Figure 5**), suggesting that Mab_A_ 1322-S0 may have acquired *erm(46)* through phage-mediated horizontal gene transfer (HGT).

## 4 Discussion

This study provides critical insights into the population genetics, recent transmission, and pan-genome of the MABC in China. Our comprehensive analysis of 360 public MABC genome assemblies, collected from eight provinces and municipalities across the country, reveals a high degree of genetic diversity and complex patterns of clonal transmission. These results underscore the importance of continuous surveillance and molecular characterization of MABC to better understand its spread, particularly in clinical settings.

ANI-based genotyping is effective for closely related bacterial strains due to its high accuracy but can be biased by incomplete genomes or size differences. In this study, we ensured accurate genotyping by applying strict quality control (genome completeness >98%, contamination<5%). Using an ANI threshold of 98%, we clearly distinguished between the Mab_A_ and Mab_M_ subspecies, with Mab_A_ comprising 75.8% of the isolates and Mab_M_ 24.2%. This subspecies distribution aligns with previous studies, which also report Mab_A_ as the more prevalent subspecies globally (Ruis et al., 2021). Phylogenetic analysis based on cgSNPs revealed significant evolutionary differences between Mab_A_ and Mab_M_, with Mab_M_ isolates exhibiting longer terminal branch lengths, suggesting a slower evolutionary rate or a more recent common ancestor. This finding is consistent with earlier research indicating that Mab_M_ is genetically distinct from Mab_A_ and may have a different evolutionary trajectory (Commins et al., 2023). The shorter branch lengths observed in Mab_A_ isolates may reflect a higher rate of transmission or adaptation to clinical environments (Ruis et al., 2021).

Our findings, consistent with previous studies, revealed genetically distinct MABC isolates, indicated by deep branches in the phylogenetic tree. Additionally, we identified highly similar Mab_A_ and Mab_M_ isolates, known as DCCs, which are globally distributed yet lack established epidemiological connections (Lipworth et al., 2021; Wetzstein et al., 2022; Olawoye et al., 2024). The proportion of DCCs in China (33.3%) is lower than in other regions of the world, such as the United States, the United Kingdom, Australia, and Malaysia, where the proportion of DCCs exceeds 50%, and in Brazil, where it reaches 100% (Davidson et al., 2013; Ruis et al., 2021). This suggests that MABC infections in China are more likely to be caused by environmentally acquired isolates, highlighting the complexity of the MABC infections in China.

Nevertheless, the detection of multiple DCCs, particularly in isolates from a single hospital in Shanghai, suggests the persistence and potential reintroduction of MABC strains over time. Given that this hospital is a major center for tuberculosis treatment in Eastern China, it likely serves patients from across the country, enhancing its national representativeness in the management of pulmonary infections in China. These findings reinforce the need for stringent infection control measures and regular genomic surveillance to prevent and manage NTM infections across China. Moreover, the identification of genomic clusters involving isolates from China and several other countries, including the UK and the USA, suggests that MABC strains may be circulating globally, likely facilitated by international travel and medical tourism (Pavli and Maltezou, 2021). This observation aligns with previous studies that have identified similar global transmission patterns in other pathogenic bacteria, such as *M. tuberculosis* (Merker et al., 2015).

This study revealed a 49.4% genomic clustering rate for MABC isolates in China, which is slightly lower than the 60% reported in the UK study and the 54.6% observed in the German study (Lipworth et al., 2021; Wetzstein et al., 2022). Additionally, we detected genomic clusters linking Chinese isolates with international strains from seven other countries, highlighting cross-national transmission (Diricks et al., 2022a). In contrast, the German study found that German isolates did not group with international strains, except within DCCs, indicating more localized transmission within Germany (Wetzstein et al., 2022). The UK study, which reported a higher clustering rate, suggested substantial recent transmission and widespread genomic clusters across a large geographical area, though it noted that person-to-person transmission in healthcare settings was not a major factor (Lipworth et al., 2021). However, these findings do not rule out the possibility of transmission in community or social settings, or from shared environmental sources within the hospital. A recent study by Commins et al. indicated that altered DNA repair and a slower mutation rate may have evolved at the base of each DCC clade, linking them to the human host (Commins et al., 2023). Therefore, the genomic study of more environmental isolates will enhance our understanding of MABC genomic diversity in its natural habitats and shed light on the behavior of environmental mycobacteria—and likely other environmental bacteria—in relation to disease causation and potential transmission to humans (Harrison and Behr, 2023).

Population genetic analysis showed that Mab_A_ has higher nucleotide diversity, and a greater rate of gene gain and loss compared to Mab_M_, suggesting more frequent genetic changes, possibly due to higher recombination rates or a broader ecological niche. The negative Tajima’s *D* values for Mab_A_ indicate an excess of low-frequency polymorphisms, hinting at recent population expansion or purifying selection, while the neutral values for Mab_M_ suggest a more stable population. Furthermore, although recombination events are less frequent than mutations, they significantly impact genetic diversity. The R/m ratio of 2.02 shows that recombination has a greater influence on MABC genomic diversity than mutation alone, consistent with findings in other free-living bacteria, such as *Bacillus cereus* ( R/m = 2.37) (Didelot et al., 2010). For comparison, the R/m ratio in *M. tuberculosis* strains varies from 0.426 to 0.565, and *M. bovis* has an R/m ratio of 0.98 (Namouchi et al., 2012; Patane et al., 2017). This genetic flexibility likely enhances MABC’s adaptability and persistence in diverse environments, including clinical settings (Bryant et al., 2021).

The antimicrobial resistance profiles of Mab_A_ and Mab_M_ in this study reveal distinct therapeutic challenges. *erm(41)*, conferring macrolide resistance, was exclusive to Mab_A_ strains, while Mab_M_ strains showed macrolide resistance through *rrl* mutations (n.2059A>G in most genomes and n.2271A>G in three). The aminoglycoside resistance-associated *rrs* mutation n.1408A>G was prevalent in both subspecies, but additional mutations (n.1355A>G, n.1375A>G) were limited to Mab_A_. Fluoroquinolone resistance-associated *gyrA* mutations (p.95D>G, p.90A>V, p.89D>N) were unique to Mab_A_, indicating its higher resistance potential and raising concerns given the importance of fluoroquinolones in treating mycobacterial infections. (Kim et al., 2018). Universally detected resistance genes, including *bla*_MAB_, *RbpA*, and *mtrA*, further underscore the multidrug resistance in both subspecies. These findings align with the high rates of antimicrobial resistance observed clinically, complicating treatment regimens in China, which rely on aminoglycosides, carbapenems, tigecycline, and macrolides (Chen et al., 2019; Griffith, 2019). Mab_A_’s broader resistance profile underscores the need for subspecies-specific surveillance and tailored therapy to combat MABC infections effectively.

The pan-genome analysis revealed a highly open pan-genome, with 79.9% of genes classified as accessory. The open nature of the MABC pan-genome suggests ongoing acquisition of new genes, which may contribute to the MABC’s ability to adapt to various environmental and host conditions (Cummins et al., 2022). The higher diversity of accessory genes in Mab_A_ compared to Mab_M_, aligns with the observed higher genetic diversity and recombination rates in Mab_A_. This finding is particularly relevant for understanding the virulence and antibiotic resistance mechanisms in MABC, as many accessory genes are likely involved in these processes (Domingo-Sananes and McInerney, 2021).

Based on our findings and previous research, the mobilome in MABC likely contributes to the strain’s genetic diversity (Dedrick et al., 2021). We identified 45 plasmids across 39 genomes, which were categorized into nine MOB clusters, with AA701 being the most prevalent. Three clusters (AA558, AE904, and AG701) were predicted to be mobilizable, suggesting potential HGT. However, no ARGs or VFs were found on these plasmids or ICEs, indicating that these MGEs may not play a primary role in resistance or virulence in MABC. This finding is consistent with recent studies showing that while MABC carries abundant and diverse prophages and plasmids, these elements are more associated with genetic diversity rather than directly driving pathogenicity or resistance (Dedrick et al., 2021; Jin et al., 2022). Specifically, we detected 783 prophage sequences, primarily from the Siphoviridae family, which could influence virulence through toxin-immunity systems (Dedrick et al., 2021). Additionally, the presence of over 700 ISs may drive genetic rearrangements and contribute to MABC’s adaptability. Overall, the mobilome in MABC is a significant factor in genetic diversity and may provide insight into its evolution and potential therapeutic targets.

Notably, the detection of the *erm(46)* gene, associated with macrolide resistance, on a prophage in Mab_A_ 1322-S0 is particularly concerning, as it suggests that MABC can acquire resistance genes through phage-mediated HGT (Bryant et al., 2021). A recent study reported a novel plasmid-mediated 23S rRNA methylase gene, *erm(55)*, which causes high-level inducible macrolide resistance in *M. chelonae* and has spread to other rapidly growing mycobacteria (Brown-Elliott et al., 2023). This finding adds to the growing body of evidence that highlights the role of prophages in the evolution of antibiotic resistance in *Mycobacteria*.

Our study, while comprehensive, has limitations. First, only the public genomic data was used, which is largely biased and commonly not well-curated. Second, the absence of environmental samples and detailed patient clinical information in our study limits the full contextualization of the molecular epidemiology results. Additionally, the MABC genomes we collected include only Mab_A_ and Mab_M_, but this does not suggest that Mab_B_ infections are absent in China. Including more geographically diverse samples, particularly global isolates, could reveal additional mixed China-global clusters and provide a clearer understanding of MABC’s global transmission dynamics. Future studies should include detailed clinical data to improve analysis robustness. Third, no experimental evidence was provided to confirm the drug resistance and horizontal gene transfer potential inferred from genomic data.

## 5 Conclusion

This study offers crucial insights into the genetic diversity, recent transmission, and resistance profiles of MABC in China. Our findings reveal significant genetic variation between the Mab_A_ and Mab_M_ subspecies, with Mab_A_ showing greater diversity and higher rates of gene gain and loss. The presence of multiple DCCs and genomic clusters containing both Chinese and global isolates highlights both local persistence and global spread of MABC. Additionally, the detection of widespread antibiotic resistance genes and mobile genetic elements, including the novel *erm(46)* gene, underscores the adaptability of MABC and the urgent need for novel therapeutic strategies. These results emphasize the importance of continued genomic surveillance and robust infection control measures to effectively manage and combat MABC infections.

## Data Availability

The original contributions presented in the study are included in the article/**Supplementary Material**. Further inquiries can be directed to the corresponding author/s.

## Supplementary material

Supplementary Table 1 Metadata for all 360 Chinese MABC genomes.

Supplementary Table 2 Subspecies identification results of all MABC strains and their ANI value to the three subspecies reference genomes.

Supplementary Table 3 cgMLST detection of MABC strains. **(A)** DCC detection results using a threshold of 250 AD; **(B)** Genomic clustering results of Chinese strains with a threshold of 25 AD; **(C)** Genomic clustering results of Chinese and global strains with a threshold of 25 AD; **(D)** Metadata of clustered global MABC genomes from BV-BRC database.

Supplementary Table 4 Detection results of MGEs in all Chinese MABC genomes, including plasmids, prophages, ISs, genomic islands, and ICEs.

Supplementary Table 5 Detection results of VFs in all Chinese MABC genomes.

Supplementary Table 6 Detection results of ARGs and resistance-conferring mutations in the genomes of all Chinese MABC strains.

Supplementary Figure 1 Characteristics of subtrees for MabA and MabM genomes. **(A)** Distribution of terminal branch lengths and **(B)** pairwise SNP distances within the MabA and MabM phylogeny. **** represents Wilcoxon rank sum test P < 0.0001. **(C)** Proportion of MabA and MabM isolates belonging to clusters (y-axis) defined by varying thresholds of maximum pairwise SNP distances (x-axis).
