# Supplementary material for: Population genetic analysis of clinical Mycobacterium abscessus complex strains in China
Source: Front Cell Infect Microbiol. 2025 Jan 20;14:1496896. doi: 10.3389/fcimb.2024.1496896 (PMC11788157; doi:10.3389/fcimb.2024.1496896)
Supplement: Supplementary Figure 1 — Characteristics of subtrees for MabA and MabM genomes. (A) Distribution of terminal branch lengths and (B) pairwise SNP distances within the MabA and MabM phylogeny. **** represents Wilcoxon rank sum test P < 0.0001. (C) Proportion of MabA and MabM isolates belonging to clusters (y-axis) defined by varying thresholds of maximum pairwise SNP distances (x-axis). [file Image1.pdf]

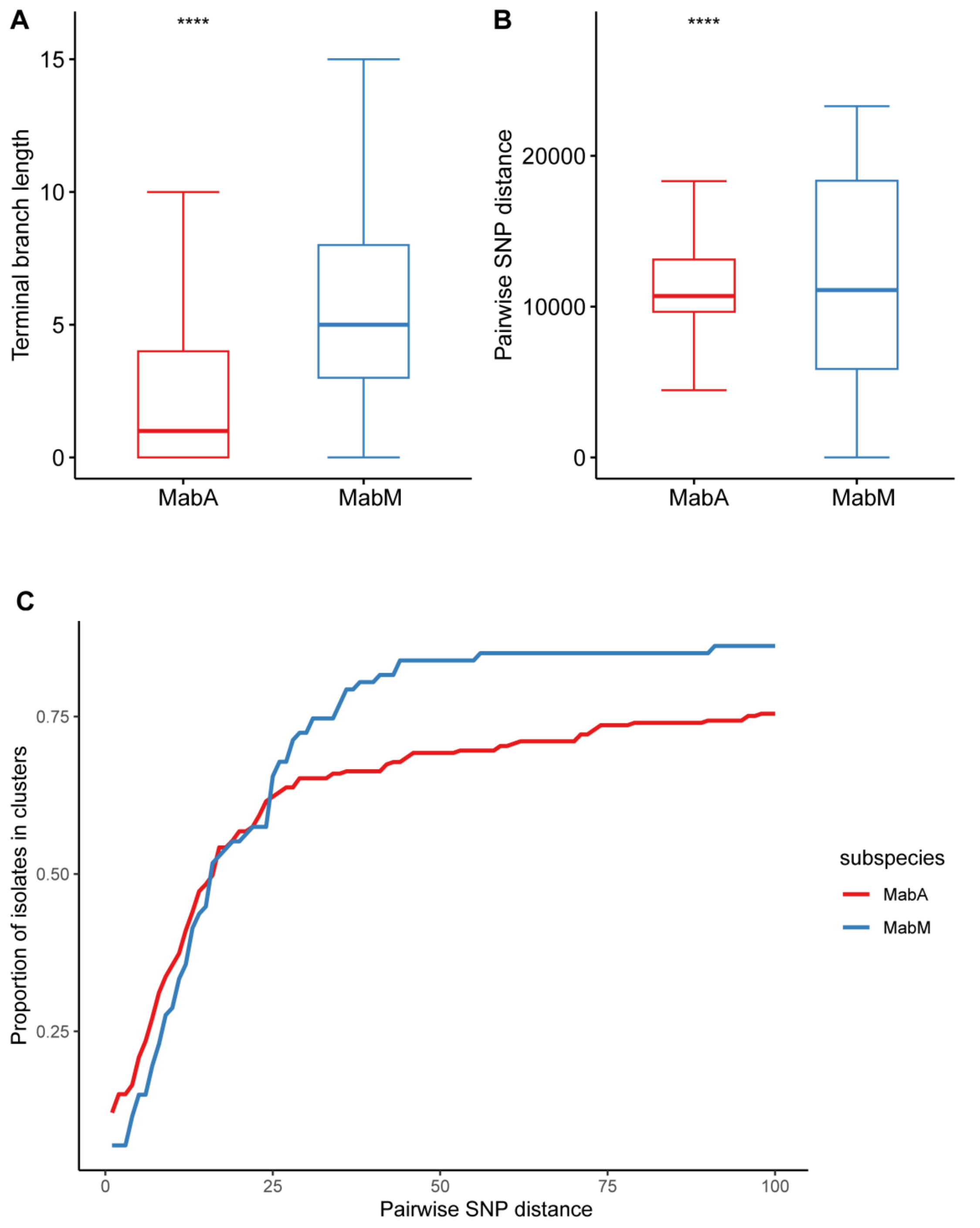

Supplementary Figure 1. Characteristics of subtrees for Mab_A and Mab_M genomes. (A) Distribution of terminal branch lengths and (B) pairwise SNP distances within the Mab_A and Mab_M phylogeny. **** represents Wilcoxon rank sum test $P < 0.0001$. (C) Proportion of Mab_A and Mab_M isolates belonging to clusters (y-axis) defined by varying thresholds of maximum pairwise SNP distances (x-axis).
